# Proceedings at the Formal Opening of the Engineering Building of the Pennsylvania State College

**Published:** 1894-03

**Authors:** 


PROCEEDINGS AT THE FORMAL OPENING OF THE ENGINEERING BUILDING OF THE PENNSYLVANIA STATE COLLEGE, February 22d, 1893. State College, Penna., 1893.

The Pennsylvania State College has had a large addition made to its imposing array of buildings by the erecting of a new edifice to accommodate the departments of Civil, Mechanical, and Mining Engineering. In 1891, the Legislature of Pennsylvania appropriated the sum of $100,000 for this purpose.

The formal opening of the building took place on February 22d, 1893, in the presence of the Governor of Pennsylvania and many invited guests.

The pamphlet under notice commemorates the event by an extended record of the proceedings and by a number of excellent illustrations of the different buildings that make up the college.

